# Implementation of research evidence in orthopaedics: a tale of three trials

**DOI:** 10.1136/bmjqs-2019-010056

**Published:** 2019-11-27

**Authors:** Katharine Reeves, Samuel Chan, Alastair Marsh, Suzy Gallier, Catrin Wigley, Kamlesh Khunti, Richard J Lilford

**Affiliations:** 1 Queen Elizabeth Hospital, University Hospitals Birmingham NHS Foundation Trust, Birmingham, West Midlands, UK; 2 Bristol Medical School, University of Bristol, Bristol, UK; 3 College of Life Sciences, University of Leicester, Leicester, Leicestershire, UK; 4 Warwick Medical School, University of Warwick, Coventry, West Midlands, UK

**Keywords:** implementation science, health policy, health services research

## Abstract

**Objective:**

To examine implementation of evidence in orthopaedic practice following publication of the results of three pivotal clinical trials.

**Design:**

Case studies based on three orthopaedic trials funded in sequence by the National Institute for Health Research Health Technology Assessment (HTA) programme. These trials dealt with treatment of fractures of the humerus, radius and ankle, respectively. For each case study, we conducted time-series analyses to examine the relationship between publication of findings and the implementation (or not) of the findings.

**Results:**

The results of all three trials favoured the less expensive and less invasive option. In two cases, a change of practice, in line with the evidence that eventually emerged, preceded publication. Furthermore, the upturn in use of the intervention most supported by each of these two trials corresponded to the start of recruitment to the respective trial. The remaining trial failed to influence practice despite yielding clear-cut evidence.

**Conclusions:**

Implementation of results of all three HTA orthopaedic trials favoured the less expensive and less invasive option. In two of the three studies, a change in practice, in line with the evidence that eventually emerged, preceded publication of that evidence. A trend or a change in practice, at around the start of the trial, indicates that the direction of causation opposes our hypothesis that publication of trial findings would lead to changes in practice. Our results provide provocative insight into the nuanced topic of research and practice, but further qualitative work is needed to fully explain what led to the pre-emptive change in practice we observed and why there was no change in the third case.

## Introduction

Evidence-based healthcare relies on clinical trials to inform practice and policy. In England, the National Institute for Health Research (NIHR) Health Technology Assessment (HTA) programme reports the findings of clinical trials. The uptake of the results of surgical trials in clinical practice can be inexpensively monitored by means of routinely collected hospital data.^1^ Over the past decade, there have been three large, multicentre randomised trials in musculoskeletal trauma in the UK funded by the NIHR HTA programme: Distal Radius Acute Fracture Fixation Trial (DRAFFT),^2^ Proximal Fracture of the Humerus: Evaluation by Randomisation (ProFHER)^3^ and Ankle Injury Management (AIM).^4^

The DRAFFT was a large, multicentre trial evaluation of volar locking-plate with the less invasive method of Kirschner wire (K-wire) fixations in the treatment for fractures of the distal radius. The study did not detect any advantage for the more invasive and expensive plate fixation.^2^ The ProFHER trial provided robust, clinically relevant evidence showing that current surgical practice does not result in a better outcome for patients with selected displaced fractures of the proximal humerus, and hence that it is not cost-effective.^3^ The AIM trial compared open reduction and internal fixation with close contact casting (CCC), for ankle fracture. This study found no advantage from surgery to counterbalance the obvious side effects and disadvantages of invasive treatment.^4^ In short, all three study findings supported use of the less invasive over the more invasive alternative. Table 1 gives an overview of the three reports considered in this paper. Each describes a randomised controlled trial comparing two treatment types for a diagnosis. The aim of HTA reports is to provide practitioners with evidence on which to base practice and policy changes. Although the reports do not make recommendations for practice, the results were clear cut in terms of the rationale for these publicly funded studies. The NIHR Collaboration for Leadership in Applied Health Research and Care (CLAHRC) West Midlands and NIHR CLAHRC East Midlands joined forces to monitor the extent to which the findings from these trials might have influenced practice.

**Table 1:** Summary of HTA study findings

Title	Distal Radius Acute Fracture Fixation Trial	Proximal Fracture of the Humerus: Evaluation by Randomisation trial	Ankle Injury Management trial
Intervention group	K-wires	Non-surgical	Surgical
Control group	Locking plate	Surgical	CCC
Age group	Aged ≥18	Aged ≥16	Aged ≥60
Trial size	461 participants recruited K-wires: 230 Locking plate: 231	250 participants recruited Surgery: 125 Conservative treatment: 125	620 participants recruited ORIF: 309 CCC: 311
Primary outcome	Patient-rated wrist evaluation questionnaire at 12 months after the fracture	Oxford Shoulder Score assessed at 6, 12 and 24 months	Olerud-Molander Ankle Score at 6 months
Secondary outcome		12-item Short Form health survey Surgical and other shoulder fracture-related complications Secondary surgery to the shoulder or increased/new shoulder-related therapy Medical complications during inpatient stay Mortality	Quality of life (as measured by the European Quality of Life 5-Dimensions, Short Form questionnaire-12 items) Pain Ankle range of motion and mobility (as measured by the timed up and go test) Patient satisfaction Radiological measures
Favoured treatment	K-wires	Non-surgical	CCC

CCC, close contact casting;HTA, Health Technology Assessment; ORIF, open reduction and internal fixation.

## Methods

### Data collection

In order to track the uptake of the treatments recommended in these three reports, we used data from the Hospital Episode Statistics (HES) database, which enabled us to plot the frequency of treatment types for specific diagnoses over time, beginning in 2003. The HES dataset contains details of all emergency and elective inpatient admissions, outpatient appointments and Accident and Emergency (A&E) attendances funded by the National Health Service (NHS) in England.

Identifying the correct diagnosis and treatment codes from the descriptions given in HTA reports proved challenging because terms used in the report are not necessarily the same as the codes used in the HES database (table 2). We sought expert advice from specialists, which enabled us to link the indication for surgery and type of surgery in HES to descriptions in the HTA report. Patients were filtered according to age and diagnosis in line with the descriptions given, and frequencies of treatment types were aggregated by 6-month intervals. After patients had been filtered by age and diagnosis, the earliest admission was retained and all subsequent admissions were excluded, in line with ‘intention to treat’ principles. Records in HES are given ICD-10 (International Classification of Disease, 10th revision) diagnosis codes and OPCS-4 (Office of Population Censuses and Surveys classification of interventions and procedures) procedure codes.

**Table 2:** Data extraction codes

DRAFFT			
Diagnosis	S52.5—Fracture of lower end of radius S52.6—Fracture of lower end of both ulna and radius		
Operation	Relevant operations in W1, W2 and W3 with Z70.5—Lower end of radius NEC		
**ProFHER**			
Diagnosis	S42.2—Fracture of upper end of humerus		
Operation	A&E	36—Sling/collar cuff/broad arm sling	
	Inpatients	Surgical	Relevant operations in W (other bones and joints)
		Non-surgical	X49.5—Application of sling NEC with one of the following: Z69.1—Head of humerus, Z69.2—Tuberosity of humerus, Z69.3—Neck of humerus
**AIM**			
Diagnosis	S82.5—Fracture of medial malleolus S82.6—Fracture of lateral malleolus S82.8—Fractures of other parts of lower leg		
Operation	Relevant operations in W (other bones and joints)		
Operation restrictions	Z85.6—Ankle joint, ie, fractures of lower leg excluded if ankle not involved		

See online supplementary appendix for more detailed codes.

A&E, Accident and Emergency; AIM, Ankle Injury Management; DRAFFT, Distal Radius Acute Fracture Fixation Trial; NEC, Not Elsewhere Classified; ProFHER, Proximal Fracture of the Humerus: Evaluation by Randomisation.

10.1136/bmjqs-2019-010056.supp1 Supplementary data

All three HTA reports specified the age of participants in the trial. In order to select patients who are as similar as possible to those in the trial, the age of patients was restricted accordingly. Patients were over 18 for DRAFFT, over 16 for ProFHER, and over 60 for AIM.

The data for the ProFHER trial (fractured humerus) starts at 2011. We did not collect earlier evidence because we have included patients who received non-surgical treatment in the A&E department, and A&E data were not included in HES until 2011.

CCC (AIM trial) is a relatively new technique used to maintain reduction of an ankle fracture. It is a modification of the total contact cast, which is regularly used to treat leg ulcers in diabetic patients.^3^ Our original plan for this analysis was to track the number of CCC treatments per 6-month interval and find whether this had risen as a percentage of the total, as in the analysis for the DRAFFT and ProFHER trials. However, it became apparent that differentiating between CCC and a traditional cast would be very difficult due to coding limitations. In an effort to identify the operation codes being used for this type of procedure, we looked at more detailed operating theatre data for University Hospitals Birmingham NHS Foundation Trust and searched for operation descriptions containing “closed contact cast”. Unfortunately, only seven procedures contained this description, all with different operation codes. Since we know that this procedure is commonly used for older patients, this is unlikely to reflect the true frequency of the use of CCC and therefore limits our ability to track it over time. We describe our approach to this limitation below.

### Analysis

After plotting the frequency of different treatment types at 6-month intervals, we calculated the frequency of the recommended treatment as a percentage of the total, as described in table 3. For the first two trials, we were able to calculate the number of procedures as a percentage of the total. However, due to the data limitations in the third trial, we were unable to calculate the total including CCC procedures, so we could only analyse the denominator-free frequency of surgical procedures.

**Table 3:** Numerators and denominators for percentages

Trial	Numerator	Denominator
DRAFFT	No of K-wire, locking plate or other procedures for patients with fracture of the lower end of the radius	Total no of procedures for patients with fracture of the lower end of the radius
ProFHER	No of surgical or non-surgical procedures for patients with fracture of the upper end of the humerus	Total no of procedures for patients with fracture of the upper end of the humerus
AIM	No of surgical procedures for patients with ankle fractures	n/a (*see above text*)

AIM, Ankle Injury Management; DRAFFT, Distal Radius Acute Fracture Fixation Trial; n/a, not applicable; ProFHER, Proximal Fracture of the Humerus: Evaluation by Randomisation.

In order to check whether there was a significant change in the frequency over time, we fitted a linear model to the time series and used a cumulative sum test to find whether this linear model fitted the data. The null hypothesis was that the cumulative sum of recursive residuals would have an expected value of 0. If it moves outside of the 95% confidence band at any point in time, it indicates that the coefficients have changed and a single linear model does not fit the data. If this was the case, we wanted to find whether the change (or break point) in the data coincided with the publication date of the HTA report. The date of the break in the model was identified using a Wald test and separate linear models were fitted before and after this date.

### Patient involvement

No patients were involved in setting the research question or the outcome measures, nor were they involved in developing plans for recruitment, design or implementation of the study. No patients were asked to advise on interpretation or writing up of results. There are no plans to disseminate the results of the research to study participants or the relevant patient community.

## Results

### Distal Radius Acute Fracture Fixation Trial

In the years 2003–2017, there were 151 951 admissions for patients who were aged 18 or over, had a fracture of the lower end of the radius and received one of the emergency treatment types of interest. Four thousand four hundred and sixty-one admission records were excluded because it was a second or higher order admission for a patient with multiple admissions, so exclusion was on an intention-to-treat basis. This brought the total number of admissions down to 147 490 (an average of 4916 every 6 months).

**Figure 1:**
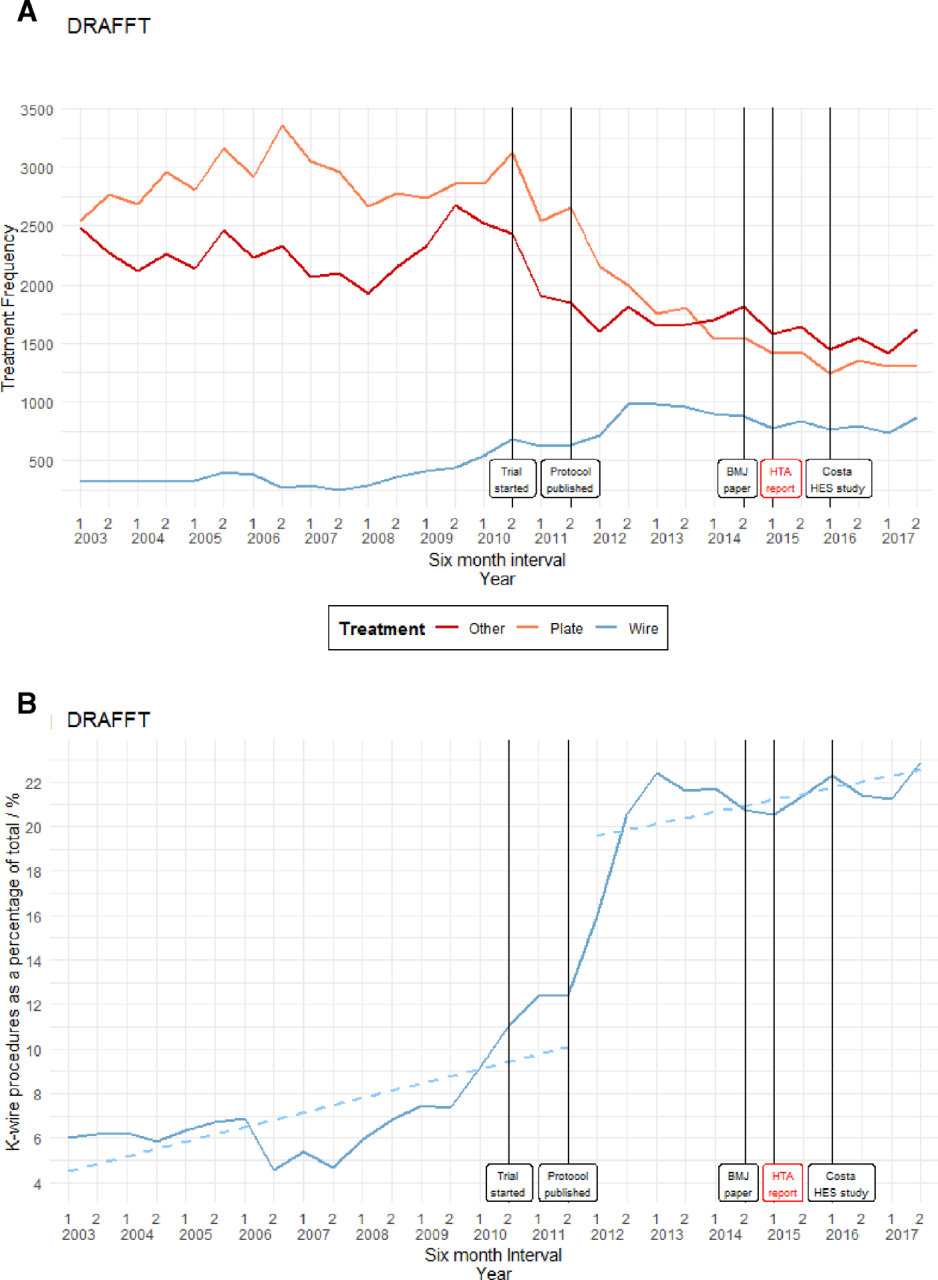
(A) Frequency of treatment types for displaced distal radius fractures: Distal Radius Acute Fracture Fixation Trial (DRAFFT). (B) K-wire fixation treatment as a percentage of the total for displaced distal radius fractures: DRAFFT. HES, Hospital Episode Statistics; HTA, Health Technology Assessment.

Figure 1A illustrates the frequency of treatments categorised as K-wire fixation, plate fixation and other. The trial began in July 2010 and the report with the findings was published in February 2015. Figure 1B illustrates the number of K-wire fixation treatments as a percentage of the total. A cumulative sum test indicates that a linear fitted model does not fit the data (test statistic=1.2858 with critical value=0.9479), and an estimated structural break corresponding to the first half of 2012 is given at the first 6 months in 2012 (p=0.000, Supremum Wald statistic=62.1275). The increase in use of the recommended treatment preceded publication of trial results and the upturn started at approximately the same time as the trial.

### Proximal Fracture of the Humerus: Evaluation by Randomisation

In the years 2011–2017, there were 13 628 admissions for patients who were aged 16 or over and had a fracture of the upper humerus. One hundred and sixty-one admission records were excluded because the patient had more than one admission with the same diagnosis, so exclusion was on an intention-to-treat basis. This brought the total number of admissions down to 13 467 (an average of 962 every 6 months). The trial began in March 2008 and the HTA report was published in March 2015.

**Figure 2:**
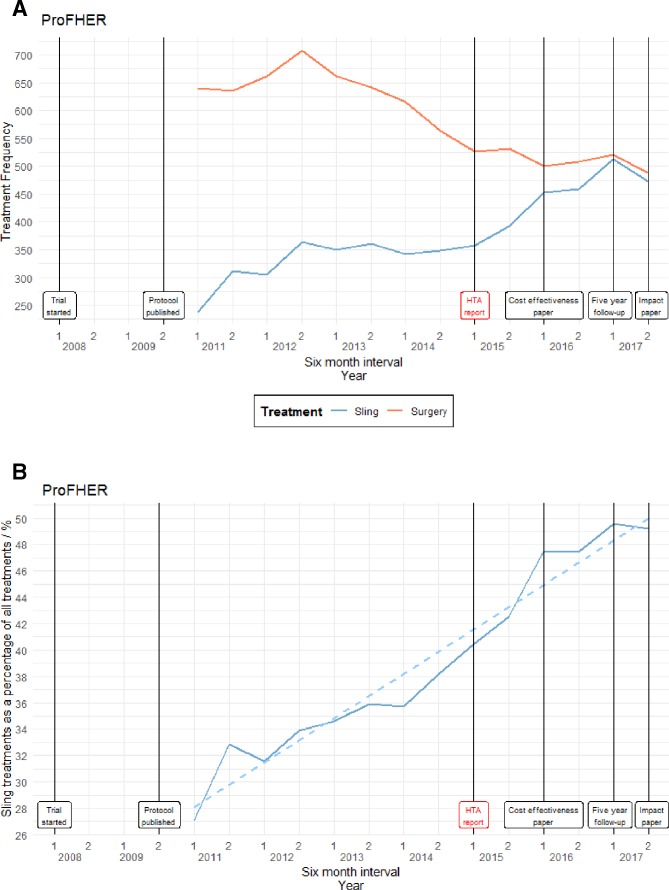
(A) Frequency of treatment types for proximal fracture of the humerus: Proximal Fracture of the Humerus: Evaluation by Randomisation (ProFHER) trial. (B) Sling treatment as a percentage of the total for proximal fracture of the humerus: ProFHER trial. HTA, Health Technology Assessment.

Figure 2A illustrates the frequency of treatments categorised as sling or surgery. Figure 2B illustrates the number of patients treated with a sling as a percentage of the total. A cumulative sum test indicates that a linear fitted model does fit the data (test statistic=0.6070 with critical value 0.9479). Although there is no clear break in the data, the less invasive procedure increased from the start of the trial, flattening off after the trial was published.

### Ankle Injury Management

**Figure 3:**
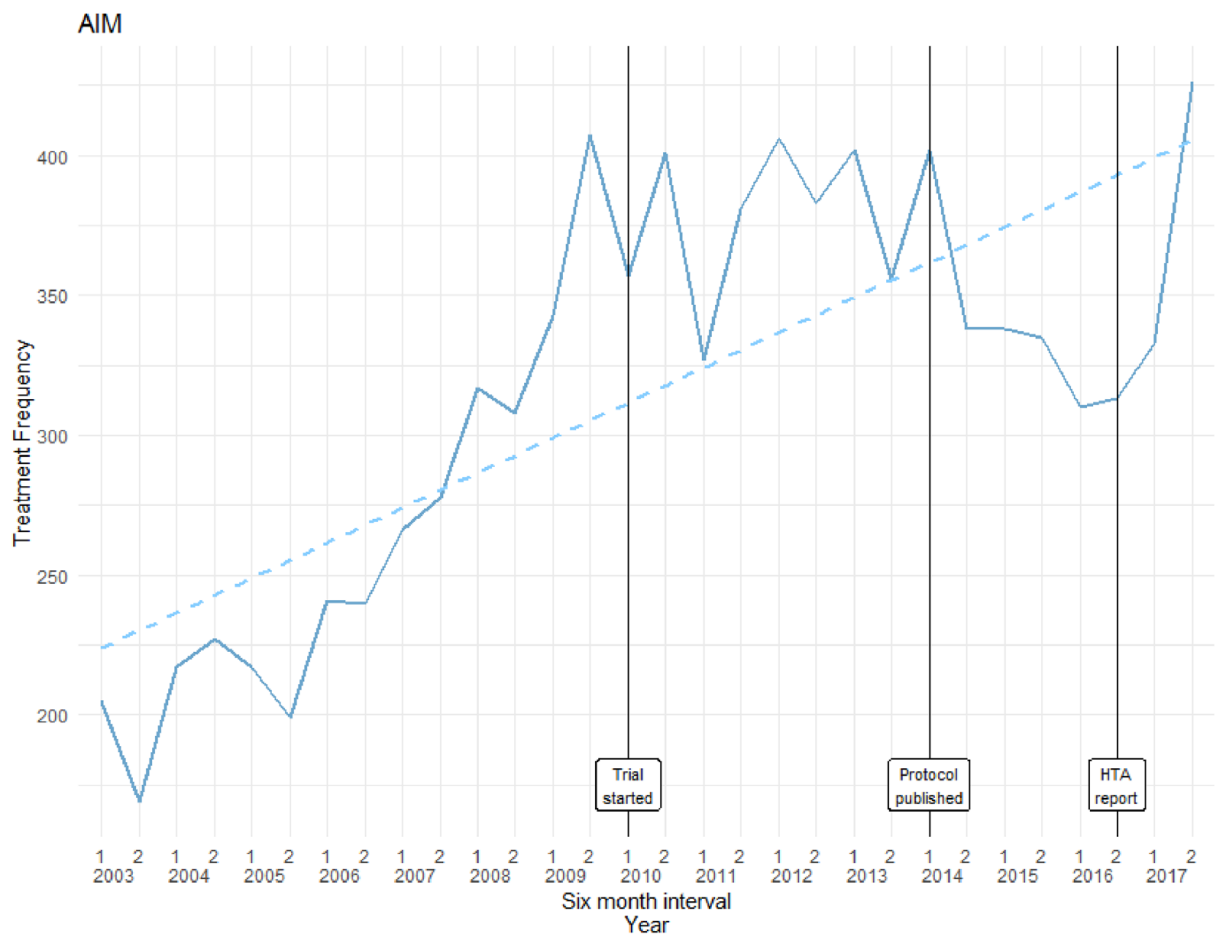
Frequency of surgery for patients aged over 60 with unstable ankle fractures: Ankle Injury Management (AIM) trial. HTA, Health Technology Assessment.

In the years 2003–2017, there were 9638 admissions for patients who were aged 60 or over, had an ankle fracture and received surgical treatment for ankle fractures of the type that were examined in the trial. Fifty-five admission records were excluded because the patients had subsequent admissions for the same diagnosis, so exclusion was on an intention-to-treat basis. This brought the total down to 9583 (an average of 319 every 6 months). The trial began in January 2010 and the HTA report was published in October 2016. Figure 3 illustrates the frequency of surgical operations. A cumulative sum test indicates that a linear fitted model does fit the data (test statistic=0.7567 with critical value=0.9479). In this study, there was no ‘anticipation’ of the results at the inception of the trial, nor does practice change in line with the published findings. Despite the finding that surgical intervention is no more effective than non-surgical care, the increase in operations seen during the trial continues after publication of the findings.

## Discussion

### Main findings

Trial results of all three cases favoured the less expensive and invasive option. In none of the three case studies did implementation of the treatment favoured by the trial increase after publication of the report. In two of the studies, a change in line with the evidence that eventually emerged preceded the publication of the findings and increased throughout the trial period.

#### Distal Radius Acute Fracture Fixation Trial

A previous review of the impact of the DRAFFT by Costa *et al* 5 used graphical summaries to find whether the frequency of each treatment type changes around the time of the trial. Unfortunately, the authors were unable to track use of the different procedures after the report publication in 2015 since their data ended in 2014. They do, however, see changes in frequency around the start of the trial in 2010 in the direction recommended by the results. It was not possible to replicate their methods because we could not replicate the codes they used. However, our findings agree with those of Costa *et al*,^5^ showing that the frequency of using a plate started to fall after 2010, with a corresponding increase in the use of K-wires.

#### Proximal Fracture of the Humerus: Evaluation by Randomisation

The recommendations made in the ProFHER report^3^ are certainly reflected in the data, with the use of slings as treatment for proximal humerus fractures consistently rising in both frequency and as a percentage of the total. However, this trend started before the start of the trial and certainly before publication of the report, implying that this may be another case where the results of the trial only confirmed the changes that were already taking place in practice. The trial reflects a growing consensus over the past few years that conservative treatment methods are preferable to surgery for many, but not all, patients with fracture of the humerus.

#### Ankle Injury Management

We were unable to track the proportion of CCC procedures among eligible patients. However, the crude surgery frequencies indicate that there is no statistically significant break in use of the procedure.

### Limitations

Our study has several limitations. It is an association study and a change in practice (in any direction) cannot be attributed to any particular causal hypothesis, beyond that showing or disproving a temporal trend. Follow-up beyond the publication of the study is short (only a year in one case). The findings from these three orthopaedic trials cannot be generalised to all orthopaedic trials, let alone all surgery trials or all clinical trials. They simply illustrate an association between the conduct of a trial and a change in practice in two case studies, and no immediate change in practice following publication of the third case.

### Failure of trial results to influence practice

A number of frameworks have been proposed to improve implementation of new knowledge resulting in adoption or abandoning a particular intervention.^6^ They start with the identification of a problem that needs to be solved. Then, if failure to implement knowledge is identified, the following steps are proposed: problem analysis (identification of barriers and facilitators); develop a theory of how to remedy the problem; work with stakeholders to develop an intervention; implement the intervention; and evaluation—a process similar to the Medical Research Council strategy for evaluation of complex interventions.^7^ Our study answers the first question—has the evidence been implemented—and does so at a national scale. The next step will be to explain why evidence obtained at considerable cost has not been widely implemented. Adapting Grol and Grimshaw,^8^ this may be because

1. The evidence was considered to be at risk of bias. These publicly funded studies were all heavily refereed prior to funding and again before publication. They were well designed but have the limitation inherent in most surgical studies that patients and surgeons were not masked to allocated group. The trials comply with the recommendations of the Balliol collaboration.^9–11^
2. The surgeons may have specific views about generalisability and applicability of results to special classes of patients. Such patient selection would be an acceptable reason for a limited change in practice overall, but it somewhat vitiates the rationale for the trial if used to explain no change in practice at all.
3. The nature of the intervention. Some interventions are more likely to lead to practice change than others, and those that require giving up a technique that one has mastered will be harder for people to relinquish than an intervention, such as a medicine, in which the practitioner may be less ‘invested’.
4. Whether the treatment is in the ‘gift’ of the clinician, or whether new funding must be provided or pathways changed. In the case of the favoured intervention in these studies, the required service disruption is minimal and the favoured treatment *less* expensive than the alternative. The exception here might be close contact casting, which requires that plaster technicians should be trained in this technique, and where cast changes are required.
5. Complex trade-offs and varying preferences creating ‘split-choice’ decisions,^12^ where some patients may have specific preferences, say to avoid the inconvenience associated with a cast.
6. Inertia. Sometimes people are ‘in a groove’ and are not opposed to change; they are just not motivated to change. Such people need a ‘nudge’, perhaps from a respected organisation, such as the National Institute for Health and Care Excellence or their Royal College. One may speculate that lack of such endorsement is a factor in the cases considered here.

### ‘Anticipation’ of study results

It would be tempting, particularly in the two cases where there was a change in practice before or at the start of the trial, to attribute the former to the latter. However, it is arguably more convincing to attribute the existence of the trial and the observed system-wide change in practice to the same ‘external’ factors. This finding, that practice across a system anticipates study results, has been described previously as a ‘rising tide phenomenon’.^13^ The ‘rising tide’ refers to a broad temporal trend where a community of practitioners come under a common influence that is both the cause of a study *and* of a more general change in practice. Orthopaedic surgeons are exposed to many influences that can explain this ‘rising tide’ effect. Discussions among surgeons in the period leading up to a trial may soften previous convictions, and the fact that the trial has been funded may provide further endorsement for an option the surgeon would not otherwise use. Orthopaedic surgeons, like other clinicians, may have become more willing to accept the less invasive of two therapies as the default procedure, as the concept of opportunity costs diffuses in society. A trial itself will always increase the use of the lesser used of any two comparator treatments, but this explanation cannot account for the magnitude of changes observed in the DRAFFT and ProFHER studies since only a small proportion of the nation’s hospitals participated in the trial.

### Next steps

The study we report here should be expanded in depth and in breadth. Depth should be extended by examining the literature for other trials/systematic reviews and by qualitative interviews to explore the reasons for findings. Breadth should be extended by tracking the correlation between the start and publication of other trials that were designed to change practice. In the meantime, our results provide provocative insight into the nuanced topic of research and practice. The results presented here show evidence of ‘anticipation’ or the ‘rising tide’ phenomenon.
