# Supplementary material for: Implementation of research evidence in orthopaedics: a tale of three trials
Source: BMJ Qual Saf. 2019 Nov 27;29(5):374–81. doi: 10.1136/bmjqs-2019-010056 (PMC7241969; doi:10.1136/bmjqs-2019-010056)
Supplement: Supplementary data [file bmjqs-2019-010056supp001.pdf]

Appendix

Table A1: Data extraction for displaced distal radius fracture (DRAFFT)

Diagnosis			
**Code**	**Description**	**Freq.**	**Perc. /%**
S52.5	Fracture of lower end of radius	188,938	84
S52.6	Fracture of lower end of both ulna and radius	35,680	16
Treatment: K-wire Fixation (Restriction: Z70.5 – Lower end of radius NEC)			
**Code**	**Description**	**Freq.**	**Perc. /%**
W24.2	Closed reduction of fracture of long bone and rigid internal fixation NEC	13,250	40
W23.2	Secondary open reduction of fracture of bone and extramedullary fixation HFQ	19,387	59
W28.1	Application of internal fixation to bone NEC	433	1
Treatment: Volar locking-plate fixation (Restriction: Z70.5 – Lower end of radius NEC)			
**Code**	**Description**	**Freq.**	**Perc. /%**
W20.1	Primary open reduction of fracture of long bone and extramedullary fixation using plate NEC	76,072	69
W24.3	Closed reduction of fracture of long bone and flexible internal fixation HFQ	15,130	14
W24.8	Other specified closed reduction of fracture of bone and internal fixation	18,395	17
Treatment: Other (Restriction: Z70.5 – Lower end of radius NEC)			
**Code**	**Description**	**Freq.**	**Perc. /%**
W10	Open surgical fracture of bone	30	<1
W12	Angulation periarticular division of bone	-	<1
W13	Other periarticular division of bone	-	<1
W14	Diaphyseal division of bone	-	<1
W16	Other division of bone	103	<1
W17	Other reconstruction of bone	-	<1
W18	Drainage of bone	-	<1
W19	Primary open reduction of fracture of bone and intramedullary fixation	12,600	7
W20	Primary open reduction of fracture of bone and extramedullary fixation	83,444	44
W21	Primary open reduction of intra-articular fracture of bone	2,231	1
W22	Other primary open reduction of fracture of bone	505	<1
W23	Secondary open reduction of fracture of bone	4,454	2
W24	Closed reduction of fracture of bone and internal fixation	34,231	18
W25	Closed reduction of fracture of bone and external fixation	3,104	2
W26	Other closed reduction of fracture of bone	49,830	26
W28	Other internal fixation of bone	143	<1
W29	Skeletal traction of bone	96	<1
W30	Other external fixation of bone	668	<1
W31	Other autograft of bone	4	<1
W32	Other graft of bone	10	<1
W33	Other open operations on bone	79	<1
W34	Graft of bone marrow	-	<1
W35	Therapeutic puncture of bone	-	<1
W36	Diagnostic puncture of bone	-	<1

Reeves K, *et al. BMJ Qual Saf* 2020; 29:374–381. doi: 10.1136/bmjqs-2019-010056

Table A2: Data extraction for proximal fracture of the humerus (ProFHER)

Diagnosis			
**Code**	**Description**	**Freq.**	**Perc. /%**
S42.2	Fracture of upper end of humerus	22,163	100
Treatment: Non-surgical (A&E)			
**Code**	**Description**	**Freq.**	**Perc. /%**
36	Sling/collar cuff/broad arm sling	2,335	100
Treatment: Non-surgical (Inpatients)			
**Code**	**Description**	**Freq.**	**Perc. /%**
X49.5	Application of sling NEC	4,145	100
Treatment: Surgical (Inpatients) (Restrictions: Z69.1 – Head of humerus, Z69.2 – Tuberosity of humerus, Z69.3 – Neck of humerus)			
**Code**	**Description**	**Freq.**	**Perc. /%**
W19.2	Primary open reduction of fracture of long bone and fixation using rigid nail NEC	2,050	13
W19.8	Other specified primary open reduction of fracture of bone and intramedullary fixation	1,204	8
W20.1	Primary open reduction of fracture of long bone and extramedullary fixation using plate NEC	10,692	68
W24.2	Closed reduction of fracture of long bone and rigid internal fixation NEC	916	6
W49.1	Primary prosthetic replacement of head of humerus using cement	71	<1
W50.1	Primary prosthetic replacement of head of humerus not using cement	24	<1
W65.4	Primary open reduction of fracture dislocation of joint and internal fixation NEC	702	4
W96.5	Primary reverse polarity total prosthetic replacement of shoulder joint using cement	24	<1

Table A3: Data extraction for unstable ankle fractures (AIM)

Diagnosis			
**Code**	**Description**	**Freq.**	**Perc. /%**
S82.5	Fracture of medial malleolus	417	4
S82.6	Fracture of latera malleolus	742	7
S82.8	Fractures of other parts of lower leg	9,015	89
Treatment: ORIF (Restriction: Z85.6 – Ankle joint)			
**Code**	**Description**	**Freq.**	**Perc. /%**
O17.2	Remanipulation of fracture of long bone and rigid internal fixation NEC	27	0
O17.8	Other specified secondary closed reduction of fracture of bone and internal fixation	13	0
W19.2	Primary open reduction of fracture of long bone and fixation using rigid nail NEC	155	2
W19.3	Primary open reduction of fracture of long bone and fixation using flexible nail	44	0
W19.4	Primary open reduction of fracture of small bone and fixation using screw	320	3
W19.5	Primary open reduction of fragment of bone and fixation using screw	257	3
W19.6	Primary open reduction of fragment of bone and fixation using wire system	46	0
W19.8	Other specified primary open reduction of fracture of bone and intramedullary fixation	460	5
W19.9	Unspecified primary open reduction of fracture of bone and intramedullary fixation	352	3
W20.1	Primary open reduction of fracture of long bone and extramedullary fixation using plate NEC	1,420	14
W20.2	Primary open reduction of fracture of long bone and extramedullary fixation using cerclage	36	0
W20.5	Primary open reduction of fracture of ankle and extramedullary fixation NEC	826	8
W20.8	Other specified primary open reduction of fracture of bone and extramedullary fixation	288	3
W20.9	Unspecified primary open reduction of fracture of bone and extramedullary fixation	130	1
W21.4	Primary intraarticular fixation of intraarticular fracture of bone NEC	34	0
W21.8	Other specified primary open reduction of intraarticular fracture of bone	7	0
W22.1	Primary open reduction of fracture of bone and skeletal traction HFQ	-	0
W22.2	Primary open reduction of fracture of bone and external fixation HFQ	24	0
W22.8	Other specified other primary open reduction of fracture of bone	15	0
W22.9	Unspecified other primary open reduction of fracture of bone	24	0
W23.1	Secondary open reduction of fracture of bone and intramedullary fixation HFQ	181	2
W23.2	Secondary open reduction of fracture of bone and extramedullary fixation HFQ	834	8
W23.3	Secondary open reduction of intraarticular fracture of bone	8	0
W23.5	Secondary open reduction of fracture of bone and external fixation HFQ	19	0
W23.6	Secondary open reduction of fracture of bone and internal fixation HFQ	335	3
W23.8	Other specified secondary open reduction of fracture of bone	7	0
W23.9	Unspecified secondary open reduction of fracture of bone	11	0
W24.2	Closed reduction of fracture of long bone and rigid internal fixation NEC	72	1
W24.3	Closed reduction of fracture of long bone and flexible internal fixation HFQ	46	0
W24.4	Closed reduction of fracture of small bone and fixation using screw	29	0
W24.5	Closed reduction of fragment of bone and fixation using screw	16	0
W24.8	Other specified closed reduction of fracture of bone and internal fixation	62	1
W24.9	Unspecified closed reduction of fracture of bone and internal fixation	25	0
W28.1	Application of internal fixation to bone NEC	37	0
W33.2	Debridement of open fracture of bone	22	0
W65.1	Primary open reduction of fracture dislocation of joint and skeletal traction HFQ	43	0
W65.3	Primary open reduction of fracture dislocation of joint NEC	235	2
W65.4	Primary open reduction of fracture dislocation of joint and internal fixation NEC	1,435	14
W65.5	Primary open reduction of fracture dislocation of joint and combined internal and external fixation	58	1
W65.8	Other specified primary open reduction of traumatic dislocation of joint	37	0
W65.9	Unspecified primary open reduction of traumatic dislocation of joint	9	0
W66.4	Primary closed reduction of fracture dislocation of joint and internal fixation	98	1
W67.1	Secondary open reduction of fracture dislocation of joint and skeletal traction HFQ	19	0
W67.3	Secondary open reduction of fracture dislocation of joint NEC	79	1
W67.4	Secondary open reduction of traumatic dislocation of joint NEC	19	0
W67.7	Secondary open reduction of fracture dislocation of joint and internal fixation NEC	1,946	19
W77.8	Other specified stabilising operations on joint	9	0
W80.2	Open debridement of joint NEC	-	0

Reeves K, *et al. BMJ Qual Saf* 2020; 29:374–381. doi: 10.1136/bmjqs-2019-010056

Table A4: Frequencies for treatment of displaced distal radius fracture (DRAFFT)

Six month interval	Frequency			Percentage of total	
	Wire	Plate	Other	Wire	Plate
2001h1	144	1,154	1,289	6%	45%
2001h2	301	2,283	2,550	6%	44%
2002h1	331	2,315	2,567	6%	44%
2002h2	338	2,577	2,558	6%	47%
2003h1	362	2,865	2,720	6%	48%
2003h2	384	3,149	2,564	6%	52%
2004h1	397	3,083	2,391	7%	53%
2004h2	393	3,381	2,539	6%	54%
2005h1	405	3,253	2,420	7%	54%
2005h2	505	3,675	2,761	7%	53%
2006h1	502	3,449	2,508	8%	53%
2006h2	372	3,931	2,654	5%	57%
2007h1	406	3,768	2,381	6%	57%
2007h2	361	3,718	2,363	6%	58%
2008h1	414	3,372	2,194	7%	56%
2008h2	548	3,653	2,535	8%	54%
2009h1	620	3,684	2,701	9%	53%
2009h2	644	3,821	3,126	8%	50%
2010h1	921	4,041	3,081	11%	50%
2010h2	1,155	4,382	2,947	14%	52%
2011h1	1,042	3,762	2,296	15%	53%
2011h2	1,099	3,962	2,339	15%	54%
2012h1	1,300	3,500	2,066	19%	51%
2012h2	1,754	3,424	2,357	23%	45%
2013h1	1,874	3,162	2,220	26%	44%
2013h2	1,797	3,116	2,131	26%	44%
2014h1	1,705	2,748	2,183	26%	41%
2014h2	1,759	2,952	2,344	25%	42%
2015h1	1,714	2,698	2,059	26%	42%
2015h2	1,760	2,715	2,100	27%	41%
2016h1	1,661	2,561	1,952	27%	41%
2016h2	1,702	2,754	2,051	26%	42%
2017h1	1,637	2,641	1,877	27%	43%
2017h2	1,818	2,707	2,114	27%	41%

Reeves K, *et al. BMJ Qual Saf* 2020; 29:374–381. doi: 10.1136/bmjqs-2019-010056

Table A5: Frequencies for treatment of proximal fracture of humerus (ProFHER)

Six month interval	Inpatients Surgery	Sling	A&E Sling	Sling Total	Surgery percentage	Sling percentage
2011h1	642	182	57	239	73%	27%
2011h2	640	179	132	311	67%	33%
2012h1	666	184	122	306	69%	31%
2012h2	707	193	172	365	66%	34%
2013h1	659	181	168	349	65%	35%
2013h2	652	215	142	357	65%	35%
2014h1	617	198	141	339	65%	35%
2014h2	565	201	147	348	62%	38%
2015h1	530	217	140	357	60%	40%
2015h2	534	248	144	392	58%	42%
2016h1	508	263	189	452	53%	47%
2016h2	514	230	232	462	53%	47%
2017h1	535	205	305	510	51%	49%
2017h2	486	258	211	469	51%	49%

Table A6: Frequency of surgery for patients over 60 with unstable ankle fractures (AIM)

Year	Six-month interval	
	1	2
2001	84	153
2002	159	159
2003	201	169
2004	218	226
2005	219	193
2006	245	235
2007	272	273
2008	313	314
2009	340	392
2010	369	398
2011	332	383
2012	405	382
2013	402	353
2014	402	338
2015	336	337
2016	314	307
2017	337	403

Reeves K, *et al. BMJ Qual Saf* 2020; 29:374–381. doi: 10.1136/bmjqs-2019-010056
